# International approaches to regulation and outcome monitoring in heart transplantation

**DOI:** 10.1016/j.jhlto.2026.100671

**Published:** 2026-09-08

**Authors:** John Onsy Louca, Beatriz Domínguez-Gil, Francisco González-Vilchez, Kiran K. Khush, Guillaume Lebreton, Christoph Knosalla, Jaime Guajardo, William Jenner, Sanjay Sinha, Maria Köhne, Clive Lewis, Antonio Rubino, Marius Berman, Jeong-Min Chun, Nicole Asemota, Wojciech Zapotoczny, Stephen Large, Sai Bhagra, Stephen Pettit

**Affiliations:** a University of Cambridge, Cambridge Stem cell Institute, Cambridge, UK; b Royal Papworth Hospital, Cambridge, UK; c Organización Nacional de Trasplantes, Madrid, Spain; d University Hospital Marques de Valdecilla, Santander, Spain; e Stanford University School of Medicine, Stanford, CA; f Hopital de La Pitié-Salpêtrière, Paris, France; g Deutsches Herzzentrum der Charité, Berlin, Germany

**Keywords:** Monitoring, Registry, Heart transplantation, Regulation, Outcomes

## Abstract

Heart transplantation improves the prognosis and quality of life in carefully selected patients with advanced heart failure. Outcomes depend on a multitude of donor, operative, and recipient factors. There is a high opportunity cost because the number of suitable donor hearts is limited. Many countries regulate their heart transplant programs, but differences in regulatory systems are not well described. We identified 39 nations that performed >20 transplants annually between 2015 and 2024. We invited transplant professionals in each nation to complete a survey on regulation in their country. Professionals from 32 nations responded, of which 28 had mandatory outcome reporting to a national registry. Outcome measures included survival (*n* = 26), primary graft dysfunction (*n* = 15), and quality of life (*n* = 5). Only 6 countries had a pre-specified definition of unacceptable outcomes after heart transplantation. Future studies might investigate the effectiveness of regulatory strategies in heart transplantation.

## Background

Heart transplantation (HTx) improves prognosis and quality of life (QoL) for carefully selected patients with advanced heart failure despite conventional treatment. Donor, operative, and recipient factors influence outcomes after HTx. Given the critical shortage of donor hearts in many countries, the costs associated with providing a national transplant program, and a population’s expectation of responsible organ utilization, there is a need to ensure acceptable outcomes in heart transplant recipients.

Regulatory systems have a role in overseeing and monitoring the delivery of transplant services through quality assurance and ensuring that national HTx services reflect the population expectations. These may identify structural challenges, including underperforming centers, and address issues that have contributed to unacceptable results. Regulation, including monitoring and transparency of outcomes, supports compliance with the World Health Organization Guiding Principles on Transplantation.^1^

We have previously summarized the regulation of HTx programs in the UK, Spain, and France.^2^ However, a wider international comparison of HTx regulatory systems has not been performed. We therefore performed a survey of HTx clinicians to understand the mechanisms through which HTx is regulated globally.

## Methodology

Nations with HTx programs with a mean number of HTx >20/year from 2015 to 2024 were identified using data from the Global Observatory on Donation & Transplantation.^3^ Transplant professionals were contacted in each nation and asked to complete a short survey (Table 1). Attending clinicians were identified through known contacts or professional networks, including the European Society for Organ Transplantation and the International Society of Heart & Lung Transplantation (ISHLT). Individuals were approached by email and invited to complete a survey designed to capture information on the basics of transplant regulation within their country. This focused survey was designed to be completed by the professional without a requirement to search for additional information. The survey was piloted on 2 transplant cardiologists in the UK to ensure questions were appropriately interpreted. Participating nations which completed the survey can be found in Supplementary Tables 1 and 2. Quantitative data were analyzed using simple descriptive statistics in RStudio-(2024.04.2+764) and are summarized in Table 1**.** Ethics approval was waived. Partaking clinicians were consented that the survey results would go towards a formal publication.

**Table 1:** Survey Questions and Responses

Section 1: General information
*Questions asked:* 1. In which country is your transplant program based? 2. What is your current role? 3. Does your nation have a national registry for heart transplantation? 4. What is the name of the regulatory organization responsible for overseeing heart transplantation? 5. Do centers have to submit outcome data to the regulatory organization?
*Focused responses:* - The roles of the individuals who completed the survey included: - Cardiologists (22) - Cardiac Surgeons (10)
*Section 2: If monitoring system present (i.e., answered yes to the last question in section 1)*
*Questions asked:* 1. If monitoring system present (i.e., answered yes to the last question in section 1). 2. Does your center submit outcome data on *** 3. Are your national outcomes available in the public domain (e.g., in medical journals or on government websites)? If “yes” - please provide a link to the most recent national outcomes. 4. Are the following outcomes subject to continuous monitoring (please select the relevant options). 5. Are outcomes externally validated? 6. Are individual center outcomes reported? 7. Is there a definition for sub-optimal outcomes? 8. How are sub-optimal outcomes defined? 9. What are the range of interventions that can be carried out for centers with sub-optimal outcomes? 10. Have any interventions had to be enacted in the last 10-year due to poor center performance? 11. How many centers have been closed in the last 10-year, if any?
*Focused responses:* Of the 28 countries that had mandatory data submission to a national registry, 16 were available in the public domain. *
The definitions of sub-optimal outcomes included: - Subjective assessment and comparison between the 2 centers. - Not meeting targets: e.g., there are national targets for waitlist survival, post-transplant survival (1 y, 3 y), etc. - Suboptimal outcomes are measured in real time using statistical process monitoring. A tabular CUSUM score of >2.5 for 90-day graft survival is unacceptable and triggers a “signal.” The tabular CUSUM step-up and step-down are derived from 90-day survival after heart transplantation in the UK. - [Survival] less than 85% in a year. - There is no single formally published national definition; sub-optimal outcomes are identified through national governance processes based on survival, graft function, and major adverse events compared with expected benchmarks. - Below a defined survival margin in-hospital, 1 and 3-years post transplantation
The number of nations which have had centers closed in the last 10-year for poor performance: No centers – 17 1 or more centers – 7 Unknown – 4
*Section 3: If monitoring systems are not present – i.e., the 4 nations that did not have mandatory reporting of data to a national registry.*
Questions asked: - If monitoring systems are not present, have any centers in your nation been closed in the last 10 years due to poor outcomes? - Are there any other ways in which outcomes are monitored - e.g., through retrospective studies of national outcomes?
Focused responses: - In the last 10 years only 1 center in 1 of these 4 nations had been closed due to poor outcomes.
The other ways in which outcomes are monitored included: - Multinational Registries. - Annual national reports performed in 2 countries.

*See the supplement for the links to the reports available from these nations.

Response to the survey.

## Results

Thirty-nine countries met the inclusion criteria for this study. Of these, clinicians from 32 countries responded to the survey (Supplementary Table 2). 22 respondents were cardiologists, and 10 were cardiothoracic surgeons. Responses are summarized in Fig. 1 & Table 1. Twenty-eight nations (88%) mandated data submission to a national regulatory organization. Twenty-six (81%) nations collected data on survival, 15 (47%) on primary graft dysfunction (PGD), 5 (16%) on QoL, and 6 (19%) collected additional data, including renal function, cardiac allograft vasculopathy, cancer, rejection, and immunosuppression. Table 2 lists the metrics published by national bodies in their annual reports.

**Figure 1:**
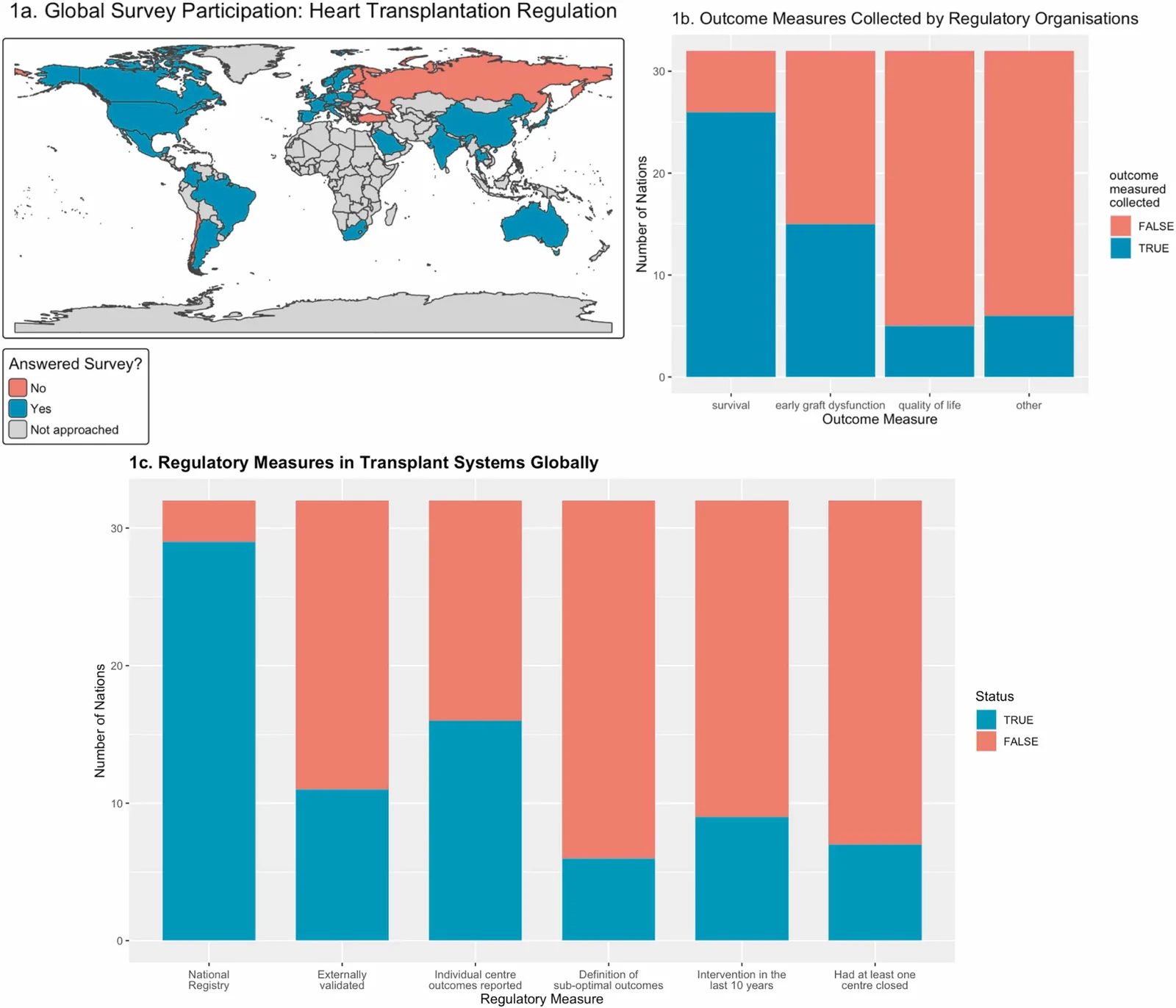
(a) *Global Survey Participation.* Nations invited to respond to the survey, categorized by response. (b) *Outcome Measures.* Number of responding nations who reported having (i) survival, (ii) early graft dysfunction, (iii) quality of life and (iv) other parameters. Other included: renal function, acute rejection, coronary artery vasculopathy, cancer, immunosuppressive regimes, and ischemic times as well as recipient and donor demographics. See Table 2 for more information. (c) *Regulatory Measures.* Number of responding nations who reported having (i) a national registry, (ii) external validation of the registry, (iii) individual center outcomes reported, (iv) a formal definition for sub-optimal outcomes), (v) who had necessitated an intervention of any sort to be implemented in the last 10 years, and (vi) have had at least 1 center closed.

**Table 2:** A Summary of the Metrics Included Annual Reports Published by Regulatory Bodies/National Societies in Close Collaboration With National Bodies

Country	Metrics Published by National Bodies	Outcomes that must be met/ Methods of identifying outliers
Brazil	Metrics by region on the number of transplants. As a nation the document includes a KM survival curve of patients from the year 2014 to 2024. No further granularity is provided.	Comparisons performed by center in each region. No risk-adjustment performed.
China	Unable to access document.	Unable to access.
France	Donor Demographics: Age Recipient Demographics: Age, Bilirubin, Blood group, Desensitization pre- and post-transplant, Diabetes, eGFR, Gender, Indication for transplantation, Induction therapy, Inotropic support pre-op, MCS use pre-op (both durable and short-term MCS), Medical care location, NT-proBNP, Region, Retransplantation, Score at registration*, Ventilation Operative Parameters: Cold ischemic timeRecipient Outcomes: Early graft dysfunction** & Survival	Risk-adjusted survival. Risk adjustment takes into account recipient age at the time of transplant, reason for transplant, MCS use at transplant, active alcoholism at the time of registration, diabetes at registration and eGFR at transplant. Donor age >55 years is also included in the risk adjustment. Survival is considered significantly different from the national average if it falls outside the 99% confidence interval.
Germany	Donor Demographics: Age, Blood group, BMI, Cause of death, Creatinine kinase levels, CK-MB level, Coronary angiography performed, Hematocrit, Hypotensive period, Organ quality at time of removal, Previous cardiac arrest, Sex, Use of vasopressors Recipient Demographics: Age, Blood group, Cardiac allocation score, Creatinine level, Diabetes, Duration between start of MCS use and admission to hospital for transplant, eGFR, Indication for transplantation, Induction therapy, PRA, Pre-transplant length of hospital stay, Previous cardiac surgery (& subtype), Pulmonary vascular resistance, Sex, Urgency status, Use of MCS pre-transplant & duration, Use of a ventilator, Viral status (Hep B, Hep C)Operative Parameters: Cold ischemic time, Organ quality at time of transplantation, Orthotopic vs Heterotopic heart transplant, Use of a perfusion system Recipient Outcomes: Cause of death, Follow-up to 1-, 2- and 3- years, In-hospital mortality, Immunosuppressive regime, Post-transplant length of stay, Reason for discharge, Retransplantation, Retransplantation on same inpatient stay, Survival, Survival is also broken down by population size for each service provider, Treated rejection episodes, Unknown follow-up status at 3-years	The report includes predefined reference ranges for hospital mortality, 1-year survival, 2-year survival, 3-year survival, and unknown follow-up status within 3 years. In provider feedback reports, analyses are performed at hospital-site level, and a site outside the relevant reference range is classified as statistically significant. The report also includes criteria for identifying cases with potential issues, primarily on data-validation criteria relating to documentation quality, plausibility, completeness, under-/over-documentation, and timely completion of follow-up.
Italy	Donor Demographics: Age, Cause of death, Donor:recipient predicted heart mismatch, Ejection fraction, Region, Sex & WeightRecipient Demographics: Age, Indication for transplant, Mutlivisceral transplant, National urgency status, Pre-transplant MCS, Previous cardiac surgery, Retransplant & Waiting time on the list.Operative parameters: Cold-ischemic timeRecipient Outcomes: Follow-up rates in first-year, Rehab post-transplantation & Survival	Funnel plots of survival with individual center outcomes plotted on the graph. The graph includes confidence intervals of 95% and 99.8% above and below the national average. Cox model for survival to compare actual center-survival to expected center-survival.
Japan	Recipient Demographics: Indication for transplant, Age, SexRecipient Outcomes: Survival, Cause of death	No comparison made between centers. Survival compared to ISHLT data in a similar time period – no risk adjustment performed.
Poland	Donor Demographics: Age, bacterial/fungal infection, Cause of death, Malignancy, Mutli-organ procurements, Region, Sex, Utilization, Viral infection (HCV)Recipient Outcomes: Early Graft Dysfunction, Region & Survival	Transplant teams are required to report significant adverse events and reactions (SAEARs) that occur during procurement, storage, allocation, or transplantation.
Spain	Donor Demographics: Age, Cause of death, Female to male transplant, Pretransplant cardiac arrest, Pretransplant echo & Sex.Operative Parameters: Bicaval technique, cold ischemic time Recipient Demographics: Age, Bilirubin, BMI, eGFR, Immunosuppressive regime, Indication for transplant, Insulin-dependent diabetes, Moderate-severe COPD, Multi-visceral transplant, Pre-op MCS, Pre-op ventilation, Previous cardiac surgery, Previous infection, Pulmonary vascular resistance, Urgency status, Use of induction therapy, WeightRecipient Outcomes: Survival & cause of death	National regulatory framework specifies requisites of centers to be authorized by regional authorities to perform HTx, including the recording of information enabling the evaluation of HTx outcomes. Centres regularly analyse internal alerts to identify potential issues. If further investigation confirms persistent deviation from expected outcomes and the underlying causes cannot be adequately addressed, the regional health care authority may revoke the center’s ability to perform HTx in serious cases.
South Korea	Donor Demographics: Age, Blood group, Cause of death, Duration of CPR & Inotrope use, Number of CPR attempts, Region & Sex Recipient Demographics: Age, Blood group, Cause of desensitization, Diabetes, HLA Crossmatch for B & T-cells, Hypertension prevalence, Indication for transplant, Multivisceral transplantation, PRA, Region, Retransplantation, Sex, Time on waiting list & Urgency status at time of transplant, Operative: Cold Ischemic time, CPB time, naso- & rectal temperatures, Operation time & Warm ischemic time Outcomes: Cardiac allograft vasculopathy, CKD prevalence, Duration on ventilator post-op, Echo at 1-month, 6-months and 1-year including LVEDD + LVESD + LVEF, eGFR, Hospitalization due to other causes, Immunosuppressive regime, Infection, Infection requiring hospitalization & associated pathogen class, MCS use post-transplant, Rejection, Retransplantation, Survival, Tumor prevalence, Use of IV medications (for heart failure – e.g. inotropes, vasopressors, diuretics & venodilators), Use of mechanical ventilation, Use of renal replacement therapy. ****	No specific mention of regulation in either of these documents.
Sweden	Donor Demographics: Age, Blood group, HCV status & SexRecipient Demographics: Age, Blood pressure, Heart rate, Height, weight, Hospitalization status, Indication for transplantation, MCS-use pretransplant, Pacemaker device inserted pre-transplant, Previous cardiac surgery, Use of renal replacement therapy & Use of a ventilator. Operative parameters: Cardio-pulmonary bypass time, Ischemic time.Recipient Outcomes: Aspirin use, BMI post-transplant, Diabetes, eGFR, Hb levels, HbA1c, Hypertension, ICU stay, Immunosuppressive regime, Infection, LDL, Primary Graft Dysfunction, Rejection, Renal Replacement Therapy, Statin use, Surgical complications, Survival, Treatment for hypertension & Unplanned readmissions to ICU.	Not mentioned in this document.
Switzerland	Donor Demographics: Age & SexRecipient Demographics: Age, Mutlivisceral Transplant, Sex & Time on waiting list Recipient Outcomes: Graft Failure, Incidence of Cancer & Survival	This report is not designed for regulatory purposes and does not serve the purpose of centre-specific outcomes comparison.
Thailand	Number of heart transplants	Document predominantly dedicated to abdominal transplantation.
UK	Donor Demographics: Age, Cause of death, Diabetes, Ethnicity, Hypertension, Hypotension, Previous cardiac disease, Sex, Smoker & Tumour, Recipient Demographics: Age, DCD/DBD donation, Ethnicity, Highest urgency during registration, In-hospital inotrope support required, In-hospital IABP support required Multivisceral transplant, Number of registrations on heart transplant waiting list, NYHA class, Previous cardiac surgery, Primary disease, Recipient sex, Serum bilirubin, Serum creatinine, Urgency at time of transplantation, Viral status (HIV, Hep B, Hep C, CMV)Operative parameters: total preservation time, Recipient Outcomes: Post-op MCS use & Survival	No specific outcomes must be met. CUSUM analysis is performed. In the annual organ specific-report there is a risk-adjusted 5-year survival for each individual centre from listing and from transplant with 95% and 99.8% upper and lower confidence intervals.
USA	Donor Demographics: Age, Blood group, Cause/ Mechanism of death, DBD/DCD donation, Distance between recipient and centre, Ethnicity, Insurance-type, Life support type pre-transplant (including MCS, inotropic support, ventilator, inhaled nitric oxide or prostaglandins) & Sex.Recipient Demographics: Age, Blood group, cPRA, Ethnicity, Indication for transplant, Multivisceral transplantation, Previous transplants, Region, Sex, Urgency status for heart recipients, VAD at transplant & Waiting time, Recipient Outcomes: Survival*****	Regulatory methods exist, but are not specifically mentioned in these documents.

A summary of the metrics included annual reports published by regulatory bodies/national societies in close collaboration with national bodies. These reports often include some form of risk adjustment to enable comparisons of individual centers.

* France uses a pre-transplant score for allocation to assess urgency.

** Defined as occurring within 24 hours post-transplant with a Left Ventricular Ejection Fraction (LVEF) < 30% and/or the need for mechanical circulatory assistance, ECMO, retransplantation, or resulting in death.

*** In Spain the Organizacion Nacional de Transplantes (ONT) does not provide an annual report. Instead, they work closely with the Spanish Heart Transplant Registry (RETC), which involves voluntary contribution of data from all centers (although all centers take part). Data is shared from the RETC to the ONT. The link to the RETC report is: https://www.revespcardiol.org/en-spanish-heart-transplant-registry-th-off-articulo-S1885585724002329. Further information on regulation can be found: https://www.boe.es/buscar/doc.php?id=BOE-A-2012–15715

**** Korean health authorities release 2 annual reports by different governmental bodies that work closely together. The first is the Korean Network for Organ Sharing (KONOS), which is the central authority for overseeing recipient selection, donor registry management, public awareness, and education campaigns. KONOS is responsible for the regulation of transplantation. In addition to the KONOS annual report, there is a more detailed Korean Organ Transplantation Registry report that is intended for use in research with the aim of improving Korean transplantation.

***** The Scientific Registry of Transplant Recipients in the US collects a vast amount of data on donor & recipient demographics and outcomes. We have not included the full data dictionary here due to its extensive length. We have only included data found in the OPTN/SRTR 2023 Annual Data Report (https://srtr.transplant.hrsa.gov/ADR/Chapter?name=Heart&year=2023#tab:HRchar-adult-tx-clin) and the Health Resource & Services Administration Data Reports (https://www.hrsa.gov/optn/data/data-reports#:∼:text=UNOS%20collects%20and%20manages%20all,return%20to%20the%20OPTN%20website) (The full data dictionary can be found here: https://www.srtr.org/requesting-data/about-srtr-standard-analysis-files-safs/saf-data-dictionary/

Five nations reported standard interventions for underperforming centers, which included warnings, audits, probations, temporary restrictions, and closure in extreme cases. There was a consensus amongst these nations that the interventions would be gradated, with more serious interventions for worse-performing programs. Eight nations closed at least 1 centre due to poor performance.

Of the 4 nations that did not submit outcome data to a regulatory organization, 2 generated a national report and 1 submitted data to a multinational registry.

## Discussion

This is the first study to summarize national regulation of HTx. There are clear differences between nations regarding regulatory processes and oversight. Most nations have some form of regulation, typically performed by a central organization. Whilst most nations monitor survival, data on other outcomes such as PGD, rejection, and QoL are less frequently collected. Most nations lack specific thresholds for sub-optimal outcomes, and only 3 nations mention the use of risk-adjustment to account for varying donor, operative, or recipient demographics (Table 2). However, given the wealth of long-term data on HTx outcomes, a definition of acceptable performance could be designed and introduced.

Differences in regulation may reflect differences in the wider healthcare system, which may reflect broader differences in healthcare systems. The need for robust system regulation may rise as a nation’s HTx volume increases. Moreover, regulation is likely to be more effective in high-volume HTx nations, as signal:noise ratio is likely to be lower in smaller HTx programs. However, it is worth noting that regulation can promote risk-averse behavior, and this has been demonstrated in other areas of clinical practice.^4^, ^5^ It is not clear whether regulation in HTx causes such risk-averse behavior, or indeed how regulatory systems should be designed to ensure appropriate risk-taking, whilst also protecting patients.

### Limitations

Our survey was not completed by 7/39 eligible nations, and therefore we are missing insight into the regulatory mechanisms in these nations. There was selection bias, as we only surveyed 1 individual per nation, and therefore we may not have captured fully accurate data about particular nations. The survey was completed by cardiac surgeons or transplant cardiologists, and this is a potential source of heterogeneity. Moreover, the survey was only sent in English, which may disadvantage respondents from nations where English is not widely spoken. Finally, no data about risk adjustment of outcomes was captured during the study.

### Going forward

Our pilot study has demonstrated considerable heterogeneity in regulatory systems, and the international community could work together to better understand the impact of regulation on heart transplant activity and outcomes. Guidance from the ISHLT on a minimum standard for regulatory systems may act as a template for nations that do not yet have these in place. Alternatively, the transplant registry could be utilized for more tailored benchmarking of post-transplant outcomes, with comparison data adjusted for baseline characteristics. Moreover, countries could be grouped by socioeconomic status, age of transplant programs and GDP per capita to ensure relevant comparisons are made. This would enable nations to compare their outcomes to a large dataset. Routine collection of standard outcome variables at standard times across nations would facilitate future research. The ISHLT thoracic transplant registries offer a wealth of data to enable such benchmarking to be performed. The utility of comparisons with this registry will increase as nations with relatively young transplant programs continue to build their contribution to the global numbers.

## Disclosure statement

JOL is supported by a grant from the Kusuma charitable trust [RCZD/069] and is the Kusuma fellow in Cardiology. JOL is supported by a grant form from the Wendy J. Tomlin-Hess fund from the Royal Papworth Charity. JOL is supported by a grant from the British Society of Heart Failure and is the BSHF Fellow. This research was funded in part by the Wellcome Trust (203151/Z/16/Z, 203151/A/16/Z) (226795/Z/22/Z) and the UKRI Medical Research Council (MC_PC_17230).

## Declaration of Competing Interest

The authors declare the following financial interests/personal relationships, which may be considered as potential competing interests: JOL is supported by a grant from the Kusuma charitable trust [RCZD/069] and is the Kusuma fellow in Cardiology. JOL is supported by a grant form from the Wendy J. Tomlin-Hess fund from the Royal Papworth Charity. JOL is supported by a grant from the British Society of Heart Failure and is the BSHF Fellow. This research was funded in part by the Wellcome Trust (203151/Z/16/Z, 203151/A/16/Z) (226795/Z/22/Z) and the UKRI Medical Research Council (MC_PC_17230).

## Footnotes

Appendix A Supplementary data associated with this article can be found in the online version at doi:10.1016/j.jhlto.2026.100671.
