# Supplementary material for: International approaches to regulation and outcome monitoring in heart transplantation
Source: JHLT Open. 2026 Sep 8;14:100671. doi: 10.1016/j.jhlto.2026.100671 (PMC13634107; doi:10.1016/j.jhlto.2026.100671)
Supplement: Supplementary file 1 [file mmc1.docx]

**Supplement: International Approaches to Regulation and Outcome Monitoring in Heart Transplantation**

**Supplementary Table. 1. Participating nations and their respective regulatory organisations.**

| Country | Regulatory Organisation | Link to most recent national outcomes |
| --- | --- | --- |
| Argentina | INCUCAI (El Instituto Nacional Central Único Coordinador de Ablación e Implante) |  |
| Australia | Donate Life | <https://anzorrg.org.au/registries/anzhtr> (no report yet, but first report expected to be published later in 2026) |
| Austria | Öbig Transplant |  |
| Belgium | Eurotransplant | <https://pubmed.ncbi.nlm.nih.gov/29492388/> (observational study with the aim of understanding outcomes of transplantation in Belgium) |
| Brazil | Sistema Nacional de Transplantes (SNT) | <https://site.abto.org.br/wp-content/uploads/2025/05/rbt-n4-2024-populacao.pdf> |
| Canada | CCTN (Canadian Cardiac Transplant Network) |  |
| China | China Organ Transplant Response System (COTRS) | https://www.tup.tsinghua.edu.cn/booksCenter/book_11037401.html |
| Colombia | National Health Institution |  |
| Croatia | Ministry of Health of the Republic of Croatia |  |
| Czech Republic | KST (Koordinačni Středisko Transplantací) |  |
| Denmark | The Health Department under the Danish government (in danish "Sundhedsstyrrelsen") |  |
| France | Agence de la Biomédecine | <https://www.agence-biomedecine.fr/fr/search/?content-types=14&content-types=32&thematiques=28&audiences=3>,  https://rams-back.agence-biomedecine.fr/uploads/2024-organe-tissus_1-14-2026_3-06-58%20PM.pdf |
| Germany | Bundesärztekammer (German Medical Association,Allocation Quality) ,G-BA (Federal Joint Comitee,outcome quality) | <https://iqtig.org/downloads/auswertung/aj2025/tx-htx/DeQS-RL_TX-HTX_AJ2025_BUAW_V02_2025-08-15.pdf> |
| India | NOTTO (National organ and tissue transplant organisation) |  |
| Italy | CNT (Centro Nazionale Trapianti) | https://www.trapianti.salute.gov.it/sites/default/files/imported/C_17_cntPubblicazioni_625_allegato.pdf |
| Japan | The Japanese Society for Heart Transplantation | https://jshtx.or.jp/wp-content/uploads/2025/08/レジストリ20241231-1.pdf |
| Mexico | CENATRA (Centro Nacional de Trasplante) |  |
| Netherlands | Dutch Transplantation Society |  |
| Norway | Oslo University Hospital |  |
| Poland | POLTRANSPLANT (Centrum Organizacyjno-Koordynacyjne ds Transplantacji) | <https://files.poltransplant.org.pl/Biuletyn_2025_www.pdf> |
| Portugal | Instituto Português do Sangue e Transplantação |  |
| Saudi Arabia | Saudi Center for Organ Transplantation |  |
| Slovakia | National Transpant Organization |  |
| South Africa | N/A |  |
| South Korea | KONOS (Korea Network for Organ Sharing) | <https://www.konos.go.kr/board/boardListPage.do?page=sub4_2_1&boardId=30> |
| Spain | Organización Nacional de Transplantes | <https://www.revespcardiol.org/en-spanish-heart-transplant-registry-th-off-articulo-S1885585724002329> |
| Sweden | Socialstyrelsen /National Board for Healt and Wellfare | <https://strax.registercentrum.se/statistik/oeverlevnad-ett-ar-efter-hjaerttransplantation/p/FoLdJHJl3>  <https://strax.registercentrum.se/om-registret/variabellista/p/ryHisi1H3> |
| Switzerland | Swiss Transplant | <https://www.stcs.ch/reports/download/STCS_Annual_Report_July2025_tpxDec2024.pdf> |
| Thailand | Thai Red Cross Organ Donation Center | <https://www.transplantthai.org/data/annual_report/13/Registry%202022%20English%20version.pdf?utm_>  source=chatgpt.com |
| UK | NHSBT (NHS Blood & Transplant) | https://nhsbtdbe.blob.core.windows.net/umbraco-assets-corp/37113/nhsbt-heart-transplantation-report-2425.pdf |
| USA | UNOS (United Network for Organ Sharing) | <https://srtr.org/>  Most recent report - <https://srtr.transplant.hrsa.gov/ADR/Chapter?name=Heart&year=2023#tab:HRchar-adult-tx-clin> |

**Supplementary Table 2. Nations that met the eligibility criteria but did not complete the survey**

Belarus

Chile

Finland

Hungary

Russia

Slovenia

Turkey
